# Super resolution DOA estimation based on deep neural network

**DOI:** 10.1038/s41598-020-76608-y

**Published:** 2020-11-16

**Authors:** Wanli Liu

**Affiliations:** Southwest China Institute of Electronic Technology, Chengdu, 610036 China

**Keywords:** Engineering, Electrical and electronic engineering

## Abstract

Recently, deep neural network (DNN) studies on direction-of-arrival (DOA) estimations have attracted more and more attention. This new method gives an alternative way to deal with DOA problem and has successfully shown its potential application. However, these works are often restricted to previously known signal number, same signal-to-noise ratio (SNR) or large intersignal angular distance, which will hinder their generalization in real application. In this paper, we present a novel DNN framework that realizes higher resolution and better generalization to random signal number and SNR. Simulation results outperform that of previous works and reach the state of the art.

## Introduction

Direction-of-arrival (DOA) estimation using multiple antenna technologies, such as smart antenna systems, is a very important problem in many applications, including wireless communications, source localization, speech enhancement, robot audition, astronomical observation, radar and sonar^1^. Signals received by the antenna arrays are down-converted into baseband signals, digitalized, and then used to form a spatial covariance matrix that is the input to most DOA algorithms. There have been long-lasting developments in DOA estimations^2^. So far, numerous algorithms have been devised to deal with the DOA estimation problem and among them, the subspace based estimation methods such as MUSIC (MUltiple SIgnal Classification) and ESPRIT (Estimation of Signal Parameters via Rotational Invariance Techniques) are well-known for their high resolution capabilities^3,4^. Performing spectral search, MUSIC can provide accurate DOA estimation at the cost of high computational complexity. Utilizing space rotational invariance, ESPRIT effectively reduces the amount of computations by avoiding spectral search. Besides, many useful modifications of MUSIC and ESPRIT algorithms have also been proposed under different conditions ^5–10^. However, these subspace based algorithms are difficult to implement in real time because of the time-consuming process of spatial covariance matrix eigenvalue decomposition.

Alternatively, machine learning approach based on artificial neural network (ANN) has also shown its potential application in DOA estimation^11–14^. Performing only basic mathematical operations and calculating elementary functions, ANNs are much faster than conventional DOA algorithms. By considering DOA estimation as a function approximation problem, ANNs show the abilities to establish the mapping between spatial covariance matrix and directions of source signals. As far as we know, the earliest attempts on ANN based DOA estimation trace back to Radial Basis Function (RBF) neural network^15,16^. RBF networks are often characterized by one hidden layer, of which each neuron is activated by one RBF (usually Gaussian function). The centers, deviations and weights of these RBFs are trained to fit a mapping between input-output pairs. The RBF networks successfully find the directions of signals, although the result is some rough. Soon, another machine learning method namely support vector regression (SVR) is proposed to deal with this problem^17,18^. SVR is based on the theory of support vector machine (SVM), which has a rigorous mathematical foundation^19^. But SVR is only suitable for small set of data and its direction finding performance is barely satisfactory. Then, multilayer perceptron (MLP) neural network is also employed in DOA estimation^20,21^. MLP neural networks are usually consisted of several hidden layers, of which the neurons are often activated by sigmoid or tanh functions. Due to the depth growth, MLP neural networks can approximate virtually any input-output mapping and are suitable for highly non-linear problems^22^. As a specialized form of machine learning, deep learning (DL) has achieved great progresses and attracted much attentions in the past few years. Different from traditional neural networks containing several hidden layers, deep neural network (DNN) often has tens or hundreds of hidden layers. With the aid of large amount of labeled data, substantial computing power and DL algorithm breakthrough, deep learning achieves state-of-the-art accuracy and sometimes exceeding human-level performance in the fields of image identification, speech recognition, natural language processing, automatic driving and so on.

Recently, some researchers have introduced deep learning techniques to solve acoustic location^23–25^ and DOA estimation problems^26–28^. There are mainly two ways of thinking about the DNN based DOA problem, and they apply to different situations. In the case of knowing the signal number in advance, the output layer of DNN has the same number of neurons, whose activation values representing the signal angles in the range of [$-\pi /2,\pi /2$]. This situation has been studied in Huang et al.’s work^26^, where the smallest location error of $0.1^{\circ }$ is realized. Apparently, the most crippling weakness of this method is the priori knowledge of signal number, which severely hinders its generalization and is also the general drawback in many previous related works. The more practical situation is that the signal number is unknown, so the DOA estimation naturally falls into a direction classification problem. In this case, the observation space is separated into *N* subspaces and the activation values of *N* neurons in output layer represent the probability of a signal locating in each subspace. This situation has been studied in Liu et al.’s work^28^, where they propose a two-stage DNN structure that is characterized by a multitask autoencoder and a group of parallel multilayer classifiers. The autoencoder denoises the DNN input and decomposes its components into *P* spatial subregions, whereas *P* parallel disconnected MLP concentrate on the direction classifications in each subregion. The autoencoder and parallel classifiers are trained independently and give the DOA estimation collaboratively with error of $0.5^{\circ }$. However, when signals are located at the edge of subregions, the corresponding DOA estimation aggravate in precision or even disappear in the estimated spectrum. Moreover, only the two-signal scenario is considered in the training process, which results in unsatisfactory performances in the scenarios of different signal number. Apart from the drawbacks mentioned above, most of the previous DNN studies locate sources on very coarse grids with spacing $5^{\circ }$^23,24^ or even $10^{\circ }$^25^. Such coarse segmentations do not meet the precision requirements in most general DOA estimation applications. Besides, in most traditional machine learning and deep learning approaches, source signals are restricted to large angular distances and same signal-to-noise ratios (SNR) in the training and validation stages, which hinders the generalization performance in real application of DOA estimation. At last, the DNN structures in all previous DOA studies are not actually deep, they are more like MLP neural networks. For the general reason that results often get better as DNN becomes deeper, we believe that a much deeper structure may boost the performance of DNN based DOA estimation.

In this paper, we take advantage of deep learning techniques to boost the resolution and generalization of DNN based DOA estimation. It is widely believed that as DNN gets deeper, its representational capacity becomes stronger and a more precise mapping function can be trained. Meanwhile, the residual technique makes it possible to train a very deep neural network in a controllable manner^29^. By considering DOA estimation as a direction classification problem, we propose a simple DNN architecture that turns out to be much better than ever before. The outline of this paper is as follows: Firstly, the data model, DNN model and training details are given in “Methods” section. Then, we show the detailed simulation results under different situations and compare with related studies in “Results” section. At last, we make the error analysis and conclude with some final remarks in “Discussion” section.

## Methods

### Data model

Let us consider a linear array composed of *L* omnidirectional antenna elements. Assume that there are *P* narrow band uncorrelated signals impinging on the array from directions $\{\theta _1,\theta _2,...,\theta _P\}$ . Under complex representation, the received signal by the *l*-th array can be expressed as

1 $$\begin{aligned} x_l(t)=\sum _{p=1}^Ps_p(t)a_l(\theta _p)+n_l(t) \end{aligned}$$

where $s_p(t)$ is the complex envelop of the p-th signal reaching antenna array at time *t*; $a_l(\theta _p)=e^{-i(l-1)2\pi d\sin (\theta _p)/\lambda }$ is the steering vector of the *p*-th signal for the *l*-th array, with *d* denoting the spacing between the elements of the array and $\lambda$ denoting the wave length; $n_l(t)$ is the background noise of the *l*-th array, which takes a zero mean complex Gaussian distribution. To avoid spatial aliasing, spacing *d* is usually set as half of wavelength $d=\lambda /2$. Equation (1) can be written in a matrix form

2 $$\begin{aligned} X(t)=AS(t)+N(t) \end{aligned}$$

where *X*(*t*), *S*(*t*), *N*(*t*) are given by

3 $$\begin{aligned} X(t)= & {} [x_1(t),x_2(t),...,x_L(t)]^T \nonumber \\ S(t)= & {} [s_1(t),s_2(t),...,s_P(t)]^T \nonumber \\ N(t)= & {} [n_1(t),n_2(t),...,n_L(t)]^T \end{aligned}$$

and

4 $$\begin{aligned} A=\left[ \begin{array}{cccc} a_1(\theta _1) &{} a_1(\theta _2) &{} \cdots &{} a_1(\theta _P) \\ a_2(\theta _1) &{} a_2(\theta _2) &{} \cdots &{} a_2(\theta _P) \\ \vdots &{} \vdots &{} \ddots &{} \vdots \\ a_L(\theta _1) &{} a_L(\theta _2) &{} \cdots &{} a_L(\theta _P) \\ \end{array} \right] \end{aligned}$$

is the array manifold matrix.

In order to perform DOA estimation, spatial covariance matrix *R* has to be determined, which is computed by the expectation value on snapshots of different time instants.

5 $$\begin{aligned} R = E[X(t)X(t)^{H}] = AE[S(t)S(t)^{H}]A^{H} + E[N(t)N(t)^{H}] = AR_sA^{H}+\sigma _n^2I \end{aligned}$$

where noises and signals are assumed to be mutually independent. Without loss of information, the upper right part of the $L\times L$ Hermitian matrix *R* can be organized into a vector of

6 $$\begin{aligned} b=[R_{11},...,R_{1L},R_{22},...,R_{2L},...,R_{LL}] \in C^{(L+1)L/2} \end{aligned}$$

As neural network does not deal directly with complex numbers, we need to extract the real and imaginary parts of each element in *b*

7 $$\begin{aligned} \bar{b}=[Re(R_{11}),Re(R_{12}),Im(R_{12}),...,Re(R_{LL})]\in R^{L^2} \end{aligned}$$

This real vector is of $L^2$-dimension because the diagonal elements of matrix *R* are purely real numbers. Then the vector $\bar{b}$ is normalized as

8 $$\begin{aligned} Z=\frac{\bar{b}}{||\bar{b}||} \end{aligned}$$

and fed into the input layer of neural network.

### DNN model

**Figure 1:**
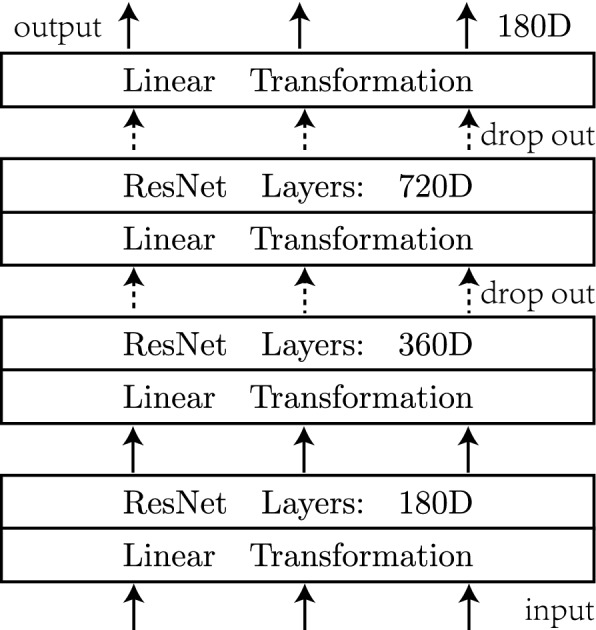
An illustration of our deep neural network. Input is transformed to vectors of larger and larger dimension via three deep residual blocks before output layer. Dashed arrows represent drop-out transforms.

The architecture of DNN used in the DOA estimation is shown in Fig. 1, which is featured by three residual blocks of the same depth. In each residual block, the linear transformation layer is used to encode data into desired dimension, and the following multilayer residual network (ResNet) is used to achieve good performance. The output layer consists of 180 neurons with sigmoid activation value representing the existence probability of signal at each angle from $-90^{\circ }$ to $90^{\circ }$. The reason for choosing larger and larger ResNet blocks is that we naively expect DNN model probe signals more clearly as dimension increases. Actually we have trained a very deep ResNet block with fixed dimension 180, but its simulation result is not so good. For our proposed DNN model here, one can also use more neurons in the output layer to improve the resolution without changing the previous body structure.

In the three-stage pipeline processing, input data *Z* is encoded into vector of more and more large dimensions. To prohibit overfitting risk and improve the generalization of our DNN model, we use dropout technique between or after residual blocks. In each residual block, linear transformation layer serves only as dimension matching. The following ResNet consist of N residual layers, and each one is composed of two linear sub-layer with hidden ReLU nonlinearity^30^ in the middle

9 $$\begin{aligned} Res(X)=ReLu(XW_1 )W_2 \end{aligned}$$

where $W_1,W_2\in R^{d_x\times d_x}$ are trainable matrices. We use the residual connections proposed by He et al.^29^ to ease the training of our DNN. So the output *Y* of each residual layer is computed by the following equation:

10 $$\begin{aligned} Y=X+Res(X) \end{aligned}$$

We then apply layer normalization^31^ after the residual connection to stabilize the activations of neurons.

In the output layer, we use the mean squared error

11 $$\begin{aligned} l_2=E\left[ ||\mathbf{u} (\theta )-\hat{\mathbf{u }}(\theta )||^2 \right] \end{aligned}$$

as the loss function, where $\mathbf{u} (\theta )\in R^{180}$ is the output of DNN and the corresponding label $\hat{\mathbf{u }}(\theta ) = \sum _i^P {{\varvec{f}}}(\theta _i)$ is actually the summation of smoothed one-hot vectors, of which the *j*-th component is set as

12 $$\begin{aligned} {{\varvec{f}}}(\theta _i)_j = 0.8\,\,if\,\,j==i\,\,else\,\,0.1*2^{-|j-i|_s} \end{aligned}$$

with $|j-i|_s$ indicating the shortest distance between *i* and *j* on the periodic interval $[-90,90]$. We find this smooth label technique also helpful to easy the training. Then, we adopt the stochastic gradient decent (SGD) algorithm to optimize the loss function based on the proposed DNN framework.

### Training details

As neural networks get very deep, the phenomena of vanishing/exploding gradients can easily take place in the training processes. To prevent this problem, a suitable weight initialization is strictly required and also beneficial to convergence rate and final performance. In our DNN model, bias is initialized to zero and weight matrix is initialized to normal distribution $W_l\sim N(0,1/d_l^2)$, where $d_l$ is the input dimension of *l*-th hidden layer. Lastly, normalization factor is initialized to one in the layer normalization operations.

Learning rate plays a key role in weight updates and convergence performance. If it is too small, the training process converges very slowly and often gets trapped in local minimums. Conversely, if learning rate is too large, the model easily diverges and results in vanishing/exploding gradients phenomena. Here we take a dynamic learning rate strategy formulized by

13 $$\begin{aligned} learning\_rate = 0.1 * min(s/s_w, \,1.0,\, (s_d/s)^2), \end{aligned}$$

where *s* is the latest training step, $s_w=200k$ and $s_d=400k$ are the warmup steps and decay-starting step. So the learning rate firstly grow linearly to 0.1, and then keeps unchanged for 200k steps, finally decay quadratically in the rest of training. This dynamic learning strategy turns out to be very efficient in our DNN training processes.

To prohibit overfitting risk, we only take use of dropout technique without L1 or L2 regularization. Dropout is applied to the outputs of second and third residual block with probability 0.5, and also applied to all residual values *Res*(*X*) with probability 0.1 in the three residual blocks. No other place is dropout involved. Because the dimension of final output layer equals that of the first residual block, we suspect dropout 0.5 following the first residual block may hinder the resolution performance. Experimental results indeed show performance degradation (not shown here) when this type of dropout is added.

**Figure 2:**
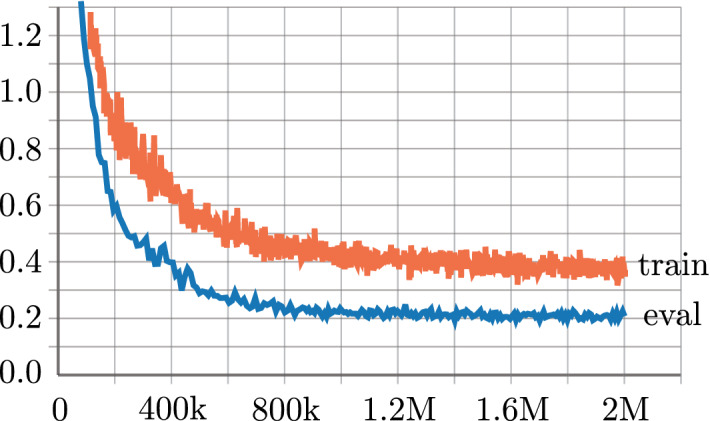
(color online) Training and evaluation losses with respect to training step.

In the training processes, data model based on 8-element uniform linear array spaced half a wavelength is used. Signal impinging angles in the training and validation sets are random in ($-90^{\circ }$, $90^{\circ }$) and ($-75^{\circ }$, $75^{\circ }$), respectively. Besides, the spatial covariance vectors *Z* in all data sets are obtained from 500 snapshots. In all the training processes for DNN of different depth, we take use of millions of samples, with batch size set as 800 and iterations as 2000k. By introducing powerful TensorFlow, the most time-consuming training process takes about 20 hours on a single GPU Nvidia Quadro P6000. Training progress of the deepest neural network is shown in Fig. 2. One can find model converge steadily and no overfitting take place.

## Results

In this section, the performances of DOA estimation based on our proposed DNN framework are investigated. Because the number of signals impinging on the array and their SNR can be varied, we carried out simulations with different settings. All simulation results are obtained from the best run after model converges.

### Fixed number of signals with equal SNR (case A)

We first investigate the performance of shallow DNN in a simple situation, which is the case of most previous studies. The training and validation sets are respectively composed of 2000k and 100k samples, carrying the information of 5 random directional signals. To show the generalization of our DNN model, signals in the training set are characterized by integer impinging angles and SNR of 10 dB, whereas in the validation set by continues floating impinging angles (deviating from integer value by 0.35$^{\circ }$ at most) and SNR of 15 dB. As a consequence, the output signal angles $\theta _{O}$ of DNN are expected as the nearest integer number of continuous input angles $\theta _I$. For comparison, we carried out experiments on DNNs of different ResNet block depth $N = 1, 3, 5$. To be strict, any estimated angle deviating from its real value by $1^{\circ }$ will be viewed as a false prediction. The corresponding estimation results of precision (P), recall (R) and their harmonic average (F-score) are shown in Tabel. 1. As one can see, the simulation performances get better as DNN becomes deeper. Lastly we state that the number of hidden layers is $6N+3$ because of three ResNet blocks and linear transformation layers, and the shallowest one is deeper than that of almost all previous ANN studies.

**Table 1:** Simulation results of case A with ResNet block depth of 1, 3, 5.

Block depth	P	R	F
$\hbox {N} = 1$	0.9251	0.9246	0.9249
$\hbox {N} = 3$	0.9469	0.9438	0.9453
$\hbox {N} = 5$	0.9562	0.9573	0.9567

### Random number of signals with equal SNR (case B)

Naturally, the number of signals cannot be previously known in most real application. So we also carried out simulations on random number of signals. The training set is composed of 4000k samples, with signal numbers random in [0,7], integer impinging angles and equal SNR of 10 dB. The validation set consists of 4 subsets with different signal numbers 1,3,5,7, respectively. Each subset is composed of 100k samples with floating impinging angles (deviating from integer value by $0.35^{\circ }$ at most) and equal SNR of 15 dB. The detailed simulation results are displayed in Table. 2. Different from the shallow DNN, a much deeper block depth is required to obtain a satisfactory result, taking the value of 5, 10 and 15 in the simulations. Apart from the improvement as the growth of ResNet depth, one can also find the performances decrease as signals become more. Comparing simulation results of five-signal subset with that of case A, unsurprisingly one can find performance get better when signal is restricted to a fixed number.

**Table 2:** Simulation results of case B on different subsets with ResNet block depth of 5, 10, 15.

Block depth	$\hbox {N} = 5$			$\hbox {N} = 10$			$\hbox {N} = 15$		
Signal number	P	R	F	P	R	F	P	R	F
1	0.9985	0.9959	0.9972	0.9991	0.9987	0.9989	0.9996	0.9994	0.9995
3	0.9894	0.9884	0.9889	0.9944	0.9934	0.9939	0.9958	0.9956	0.9957
5	0.9259	0.9203	0.9231	0.9350	0.9300	0.9325	0.9409	0.9406	0.9408
7	0.8131	0.7788	0.7956	0.8289	0.8013	0.8149	0.8379	0.8226	0.8302

**Figure 3:**
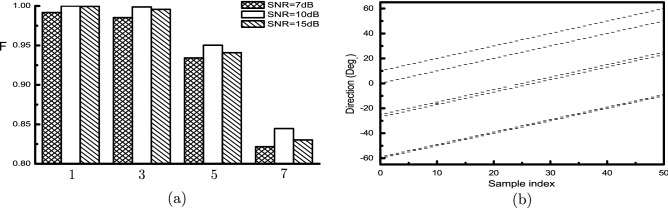
(**a**) Validation performances of 4 subsets with different SNR. (**b**) DOA estimations of 6 signals with $\hbox {SNR}=10\,\hbox {dB}$.

Moreover, we show the simulation results under different SNR as in Fig. 3a, where SNR influences are clearly displayed with respect to signal number. Naturally, the best results belong to the 10 dB validation set that is equaled to the training set. Performance declination of 7 dB set is larger than that of 15 dB, which is attributed to relatively increased influence of noises. Finally, we give an illustration of DOA estimation for this case. Six signals of 10 dB are assumed to impinge onto antenna array simultaneously, with initial angles $[-60^{\circ },-59^{\circ },-27^{\circ },-25^{\circ },0^{\circ },10^{\circ }]$. Keeping the angular distances unchanged and evolving the first signal from $-60^{\circ }$ to $-10^{\circ }$ with a step of $1^{\circ }$, we show the direction estimation results in Fig. 3b. Only one false-dismissal and one false-alarm occur for the third and forth signals, resulting in a very high F-score 0.9967 for this example.

### Fixed number of signals with random SNR (case C)

Previous works on ANN-based DOA estimation are mainly focused on the condition of equal SNR, so we test the performance of our DNN-based model on the condition of random SNR. The training and validation sets respectively consist of 4000k and 100k samples, both characterized by 4 signals of integer impinging angles and random SNR between 5 and 20 dB. In the simulations, we find the expected signals of small SNR often missed, which causes large recall decrease and is faithfully displayed in Table. 3. Because of the sharp drop compared with case B and limited improvements as the growth of DNN depth, we do not go further on the case of ‘random number and random SNR’. Nevertheless, this result is still acceptable.

**Table 3:** Simulation results of case C with ResNet block depth of 5, 10, 15.

Block depth	P	R	F
$\hbox {N}=5$	0.9390	0.8664	0.9013
$\hbox {N}=10$	0.9382	0.8779	0.9071
$\hbox {N}=15$	0.9376	0.8814	0.9086

### Direction estimation resolution

**Figure 4:**
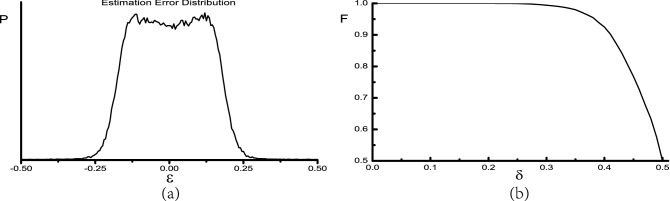
(**a**) Estimation error distribution of two signals with $\hbox {SNR}=10\,\hbox {dB}$. (**b**) F-score of two 10 dB signals with respect to direction deviations from integer impinging angle.

The idea of direction classification is very useful when signal number is unknown, but it has a major defect: the higher resolution required, the larger class number and bigger neural network are needed. To overcome this problem, we take the method of amplitude interpolation to estimate signal directions of non-integer impinging angles, which is expressed as

14 $$\begin{aligned} \theta _I = I + (u_{I+1}-u_{I-1}+2u_{I+2}-2u_{I-2}+3u_{I+3}-3u_{I-3})/0.8 \end{aligned}$$

where $\theta _I$ is the estimated direction of the *I*-th peak of neural network output $\mathbf{u} (\theta )$, and $u_{I\pm n}$ is the neuron activation amplitude of the *n*-th neighbour. For example, the distribution of estimation error for case B is shown in Fig. 4a, where the proportion of different error $\varepsilon$ is counted from 10k samples of two signals with SNR=10 dB. As one can see, almost all errors are smaller than $0.25^{\circ }$. Statistically, the average and standard derivation of absolute estimation error is $\mu =0.1^{\circ }$ and $\sigma =0.06^{\circ }$, respectively. Through this interpolation method, performance of DOA estimation is largely improved.

Alternatively, there is another perspective to investigate estimation error. When impinging angles deviate from integer values by $\delta =0.5^{\circ }$, the direction classification will be ambiguous. So we expect performance declination of direction classification as $\delta$ increases gradually from $0^{\circ }$ to $0.5^{\circ }$. As is shown in Fig. 4b, F-score of direction classification falls from 1.0 at zero deviation down to 0.5 at middle point. Estimation error starts to pull down F-score greatly when it fills the gap between $\delta$ and middle point, formulized as $\delta +\mu +\sigma \approx 0.5^{\circ }$. So we can find the sharp collapse near $\delta =0.35^{\circ }$, which is in good agreement with the straight estimation error distribution.

### Comparison with previous studies

We compare our results with two latest DNN studies on DOA estimations carefully. In Liu et al.’s study^28^, the training process is restricted to two-signal scenario and the validation process is carried in the situation of big intersignal distance ($9.4^{\circ }$). When their result is generalized to three-signal scenario, the estimation errors will grow dramatically even though a larger intersignal distance ($14^{\circ }$) is applied. What is worse, because of their subregion technique, DOA estimation performance becomes terrible when signals are located at the edge of subregions. In Huang et al.’s study^26^, the most crippling weakness is that the signal number must be known in advance, which severely hinders its generalization in real applications. Although our DNN structure is fairly simple, it outperforms that of previous works; see Table. 4. Firstly, our DNN model is suitable for random number of signals, or rather, no priori knowledge of signal number is needed. Secondly, compared with Liu et al.’s work, our model is not troubled by the subregion edge aggravation problem and applicable in a larger spatial region (smaller than Huang et al.’s, but it is good enough in real application). Thirdly, our model keeps a very high resolution with the least number of antenna elements in a generalized condition. Although we just reach the same resolution as Huang et al in our simulation, it can be easily enhanced if we increase the element number of antenna array (this is well known) or enlarge the output size of our DNN model. Lastly, most previous works are restricted to zero or small SNR differences in validation processes, whereas our model is still nearly applicable in the case of large SNR difference. In general, we can safely say that we have reached the state-of-the-art DNN-based DOA estimation.

**Table 4:** Comparison with previous studies on DOA estimation based on neural networks.

Model	Validation signal angular distance	Spatial scope	Estimation error	Signal number	Max SNR difference	Antenna number
Liu et al.^28^	$9.4^{\circ }$	$[-60^{\circ }, 60^{\circ })$	$0.5^{\circ }$	Fixed	3 dB	10
Huang et al.^26^	Random	$[-90^{\circ }, 90^{\circ })$	$0.1^{\circ }$	Fixed	0 dB	128
Our DNN	Random	$[-75^{\circ }, 75^{\circ })$	$0.1^{\circ }$	Random	15 dB	8

## Discussion

In the previous simulations, the integer-float angle inconsistent between the training and validation sets will hinder the performance of DOA estimations. We find that if the validation set changes to the one of integer angle, the F-score will obtain improvements of at most 2.0 absolute percentages. Apart from that, estimation errors come from three aspects: neighboring signals, large angles and SNR differences.

Firstly, we find that one input signal may split into two output neighbors or two neighboring input signals may fuse into one output. For example, input signal of angle $50^{\circ }$ sometimes generates prediction of two signals of angle $50^{\circ }$ and $51^{\circ }$, which causes the precision decrease. Conversely, input signals of angle $34^{\circ }$ and $36^{\circ }$ sometimes fuse into one output of angle $35^{\circ }$, which causes both precision and recall decrease. As signals become more and more, the probability of neighboring occurrences gets larger. So we can see the performance decrease as the increasement of signal number in the above simulations. Furthermore, in the process of amplitude interpolation estimations, amplitude peaks of neighbouring signals will interact with each other greatly, making our interpolation method inaccurate (maybe there exist other proper amplitude interpolation methods, but the final estimation error will not be greatly reduced).

Secondly, ANNs obtain the abilities to establish the mapping between input features and output predictions after training. In our proposed DNN, the mapping function between input steering vector and output angles can be symbolically written as

15 $$\begin{aligned} \theta _O=f(\sin \theta _I) \end{aligned}$$

To generate the right predictions $\theta _O=\theta _I$, the mapping function *f* is expected as arcsine, whose gradient $f^\prime (\sin \theta _I)=1/\cos \theta _I$ approaches infinite near large angle $\pm 90^{\circ }$. As for neural networks, it is easy to fit a smooth function but hard to fit a very sharp one. So the mapping function *f* will deviate from arcsine at large angles and result in prediction errors. That is why we test our DNN model in ($-75^{\circ }, 75^{\circ }$) but not the whole interval ($-90^{\circ }, 90^{\circ }$).

Lastly, situation of different SNR has larger variable space compared with that of equal SNR, making it difficult to fit a precise mapping function. Furthermore, signals of higher SNR tend to cover up the ones of lower SNR. The extraction of weak signals is a general challenging problem in DOA estimations.

In conclusion, an efficient DNN-based model for superhigh resolution DOA estimation is proposed in this paper. The key advantage of ANN-based models over conventional subspace based methods is the ability to provide accurate DOA estimations almost instantaneously as they avoid complex matrix calculations. Compared with other ANN-based models, our DNN model has much deeper depth and keeps higher resolution in more general conditions, such as random signal number, random SNR and small angular distances. However, this model does not work very well in the condition of large signal number, large SNR difference or neighboring signals. This inspires us that ResNet is not adequate, and some other techniques are still required. For example, stacked autoencoders^32,33^ or restricted Boltzmann machines^34,35^ may be useful in signal denoising. Specially, convolutional neural network (CNN)^36^ may be more efficient to estimate signal directions from spatial covariance matrix *R*, whose geometry features may be lost in present DNN input *Z*. A more general DNN-based algorithm of superhigh resolution in the condition of random signal number and random SNR is left for a future work.
